# Uterine leiomyoma associated non-puerperal uterine inversion misdiagnosed as advanced cervical cancer: A case report^☆^

**DOI:** 10.1016/j.ijscr.2013.08.011

**Published:** 2013-09-08

**Authors:** Osita Samuel Umeononihu, Joseph Ifeanyi Adinma, Nworah J. Obiechina, George Uchenna Eleje, Onyebuchi Izuchukwu udegbunam, Ikechukwu Innocent Mbachu

**Affiliations:** Department of Obstetrics and Gynecology, Nnamdi Azikiwe University Teaching Hospital, Nnewi, Anambra State, Nigeria

**Keywords:** Chronic uterine inversion, Non-puerperal, Tumour, Misdiagnosed, Gynaecological near miss

## Abstract

**INTRODUCTION:**

Uterine inversion is an un-common complication of parturition which often occurs in the immediate postpartum period. The chronic (non-puerperal) uterine inversion is rarer and most times tumour associated.

**PRESENTATION OF CASE:**

A 51-year old grand multiparous lady presented with a month history of abnormal vaginal bleeding associated with offensive vaginal discharge, lower abdominal pain and dizziness. The initial evaluation suggested severe anaemia secondary to advanced cervical cancer. Examination under anaesthesia (EUA), staging and biopsy was attempted but this was however inconclusive due to profuse haemorrhage. A repeat EUA revealed chronic uterine inversion secondary to fundal submucous uterine leiomyoma. Myomectomy was done with tissue histology confirming benign uterine leiomyoma. Two weeks later, a modified Haultain's procedure was done followed by simple hysterectomy and posterior colpoperineorrhaphy. She had satisfactory recovery.

**DISCUSSION:**

This is the first reported case of chronic non-puerperal uterine inversion in our hospital. When it occurs, it is usually tumour associated with the commonest tumour being prolapsed myoma and leiomyosarcoma. The diagnosis is based on high index of suspicion.

**CONCLUSION:**

Chronic uterine inversion is a rare gynaecological condition and can be misdiagnosed as advanced cervical cancer or other causes of severe genital haemorrhage in women. A high index of suspicion is needed for its proper diagnosis. Sometimes, an EUA and biopsy was required to determine the cause here and conveniently it could be described as a “gynaecolological near miss”.

## 1 Introduction

Uterine inversion is an un-common complication of parturition which often occurs in the immediate postpartum period.^1–3^ The chronic (non-puerperal) uterine inversion is rarer and most times tumour associated.^4,5^ The diagnosis of non-puerperal uterine inversion is often difficult and requires high index of suspicion.

## 2 Presentation of case

A 51-year old para 7 + 0 (with six living children) Igbo Nigerian woman, a petty trader and could not ascertain her last normal menstrual period, presented with abnormal vaginal bleeding of one month duration. This was associated with offensive vaginal discharge, lower abdominal pain and dizziness. She had no associated abdominal mass and she also had no history of significant weight loss. She had history of menorrhagia in her late reproductive years which stopped about 3 months prior to presentation which she attributed to menopause. She had no prior history of PAP smear test. She is not a known hypertensive or diabetic and she had no bowel symptoms but she had dysuria and frequency.

The physical examination showed an anxious and markedly pale woman. Her pulse rate was 108 beats per minute (moderate volume and regular). The blood pressure was 100/60 mmHg. The abdomen revealed a midline sub-umbilical scar that healed primarily otherwise there was nothing remarkable. The vulva pad was heavily soaked with fresh blood. The vagina harboured a fungating and friable mass that almost filled the cavity obscuring the cervix. Further examination was not possible on account of haemorrhage.

The investigations results were: packed cell volume of 19% and urinalysis showed numerous white and red blood cells, protein and bacteria debris. The HIV serology was negative. Abdomino-pelvic ultrasound showed dilated cervical canal harbouring hypoechoeic fluid with low level echoes in its cavity. It measured 7.5 × 6.9 × 9 cm. There was thickening of the endometrium with fundal indentation which the sonologist thought suggested arcuate uterus. There was no enlarged node seen and the other intra-abdominal organs were sonologically normal. The sonologist's impression was: cervical stenosis secondary to cervical cancer. He also queried uterine anomaly. A diagnosis of anaemia secondary to advanced cervical cancer was made.

The resuscitation/stabilization was initially with crystalloids until blood was available. Eight units of red cells were transfused. Broad-spectrum antibiotic therapy was commenced. She was counselled for examination under anaesthesia (EUA), staging and biopsy.

**Fig. 1:**
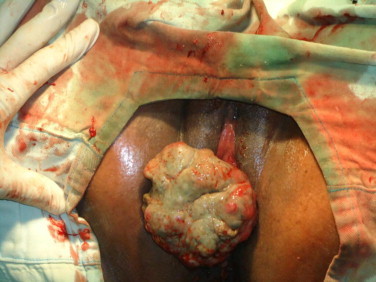
Large myoma abutting from the uterine fundus.

The initial attempt at EUA was unsuccessful due to provoked torrential haemorrhage. However a repeat by the senior consultant revealed a firm exophytic sessile mass (6 × 8 cm) with necrotic surface abutting from the inverted uterine fundus (Fig. 1). The cervix was visualized constricting the superior margin of the mass. The uterus was about 14 cm size with dimpling of the fundal margin. There was no anterior wall prolapse.

We made a diagnosis of chronic non puerperal uterine inversion secondary to submucous fibroid in a grand multiparous woman. The fibroid mass was excised haemostasis achieved ligation and diathermy cautery. The mass was sent for histology which confirmed it as benign myoma. The histology report is as shown below:

Macroscopy revealed greyish white to tan firm to hard tissue measuring 6.5 × 3.0 × 7.0 cm. Cut section showed greyish-white to tan with whorled appearance and a cystic space measuring 2.0 cm in its widest diameter. Microscopy sections showed benign mesenchymal neoplasm composed of proliferating matured smooth muscles disposed in whorls and interlacing fascicles. There were focal areas of ulceration and granulation tissue formation. Overall features are those of ulcerated leiomyoma. The histology result was discussed with the patient and she was seen two weeks later for reduction under conscious sedation. This was unsuccessful and patient counselled for operative reduction but she rather preferred hysterectomy.

**Fig. 2:**
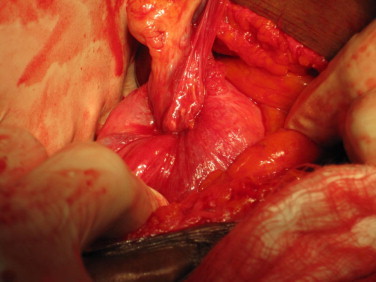
Laparotomy view of the uterus before correction of inversion. Note the characteristic dimpling and in-turning of the fallopian tubes and round ligaments.

**Fig. 3:**
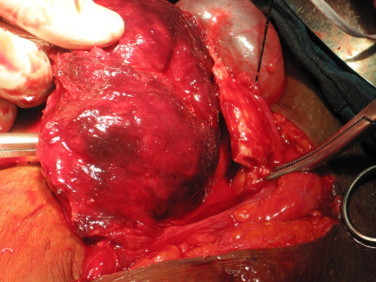
The uterus showing the endometrial surface after reversion.

**Fig. 4:**
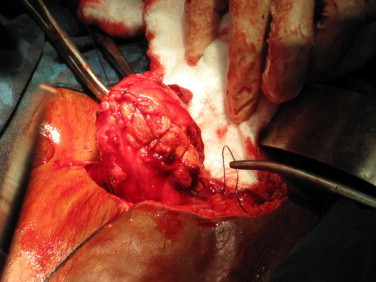
The repaired uterus before the simple hysterectomy.

The operation of uterine inversion and simple hysterectomy was done. The findings included: Mild intra peritoneal adhesions, inverted uterus with marked fundal dimpling. The endometrium had patchy ulcerations and bled on contact. The right ovary appeared normal while the left harboured a simple cyst. The infundibulo-pelvic, ovarian and round ligaments were in-turned and stretched as well as the fallopian tubes (Fig. 2). The constricting cervical ring was assessed by insinuating lens tissue forceps through the dimple and opening it out (the author's procedure). The blade borne Badparker was gently insinuated to the ring and cut through the full thickness of the ring anteriorly avoiding the bladder (the author's procedure). Once this was done, the edge was picked by another Lens forceps and pulled out to reverse the inversion (Fig. 3). The longitudinal anterior uterine incision was sutured to maintain the uterine anatomy (Fig. 4). This also minimized bleeding while completing the hysterectomy according to standard procedure. The surgery was completed vaginally with posterior colpo-periniorrhaphy.

## 3 Discussion

This is the first reported case of chronic non-puerperal uterine inversion in our hospital, which is sited at the south-east part of the country. The other previous reports in Nigeria were in south–south region of the country.^4,5^

Chronic non-puerperal uterine inversion is a rare finding in women with less than 200 cases reported in the literature since 1887.^1,2^ When it occurs, it is usually tumour associated with the commonest tumour being prolapsed myoma and leiomyosarcoma.^3–8^ The patient we managed had tumour associated chronic inversion in which the implicated tumour was a fundally sited sessile fibroid.

Uterine inversion refers to a descent of the uterine fundus to or through the cervix, so that the uterus is turned inside out.^6^ It can be acute or chronic. Acute uterine inversion causes severe pain and haemorrhage and almost always an aftermath of delivery whereas chronic inversion is insidious and characterized by pelvic discomfort, vaginal discharge, irregular vaginal bleeding and anaemia.^8–10^

The diagnosis is based on high index of suspicion. The clinical features may include: lower abdominal tenderness, vaginal bleeding, urinary frequency, dysuria and urgency. Finding a mass coming through the cervix without definite margins of a cervix, absence of the uterine fundus or fundal dimpling during bimanual or rectal examinations are strongly suggestive of the diagnosis. The openings of the fallopian tubes may be identifiable if it had been dragged through the endometrial surface. The diagnosis is easier with complete inversion when a bluish-red mass is identified from the vulva with a constricting ring of the cervix superiorly.^9^ In other cases, the diagnosis can be very difficult.

Imaging procedures such as ultrasound and magnetic resonance imaging will assist the diagnosis. Unfortunately, because of the rare nature of the disorder, uterine inversion frequently goes undetected until surgery unless a high index of suspicion is maintained. Ultrasound examination is the first line imaging investigation.^7,8^ The suggestive features include: indentation of the fundal area and depressed longitudinal grove extending from the uterus to the centre of the inverted uterus. When available, Magnetic Resonance Imaging (MRI) can be very useful.^9^ The features will include: “U” shaped uterine cavity, thickened and inverted uterine fundus on sagittal section and “bull's eye” configuration on an axial Image.^9^ Some authors have recommended the use of T2-weighted MRI.^9^ Frozen section of the vaginal mass has been used by some authors for the diagnosis. Demonstration of the endometrium on the surface of the mass will confirm the diagnosis. Biopsy of the mass has definite place if an associated malignancy is suspected as in our index patient.

In general, treatment is guided by whether condition is acute or chronic, reproductive wish of patient and cause of the inversion (benign or malignant condition). Many surgical methods (vaginal and/or abdominal) have been described to treat non-puerperal uterine inversion.^10^ The efficacy of the nonsurgical methods is not clear. Most of the surgical methods described involve reinverting the uterus before both repairing the incisions made and proceeding to hysterectomy or outright hysterectomy. Vaginal hysterectomy without reinverting the uterus has been reported. Repositioning of the uterus is usually done after the tumour has been removed and malignancy must be excluded.

Surgery is imperative in chronic inversion unlike in acute inversion where manual repositioning is possible. Depending on the clients reproductive desire and associated conditions, surgical reposition or hysterectomy could be done through either abdominal or vaginal approach.

Spinelli and Kustner are similar trans-vaginal surgical reposition techniques with the basic differences being that Spinelli's approach is anterior and requires dissection of the bladder and an anterior uterine wall incision, while Kustner's is a posterior approach with incision on the posterior uterine wall, which makes it a bit easier and safer.^10^

However, surgical repositioning can also be done through a laparotomy using the Huntington procedure, which consists in locating the cup of uterus formed by the inversion, dilating the cervical ring digitally, and gentle upward traction of the round ligaments of the uterus.^2,3,10^ The Haultain procedure uses a vertical incision in the post portion of the ring and gentle traction on the round ligaments. We used the modified Haultain procedure for our patient because it was technically easier for us.

Nevertheless, in places where facility and expertise exist, this repair could be done laparoscopically.^11^ Auber et al.,^11^ described a case of non-puerperal uterine inversion using combined laparoscopic and vaginal approach. This method of approach is now emerging from available literature.

## 4 Conclusions

Chronic uterine inversion is a rare gynaecological condition and can be misdiagnosed as advanced cervical cancer or other causes of severe genital haemorrhage in women. A high index of suspicion is needed for its proper diagnosis. Sometimes, an EUA and biopsy was required to determine the cause here and conveniently it could be described as a “gynaecolological near miss”.

## Conflict of interest

The authors declare that they have no competing interests.

## Funding

The authors alone are responsible for the contents and writing of the paper. They have conducted this study in the course of their service in the hospital and have not received funding from any organization.

## Ethical approval

Written informed consent was obtained from the patient for publication of this case report and any accompanying images. A copy of the written consent is available for review by the Editor-in-Chief of this journal on request.

## Authors’ contributions

SOU and GUE performed the surgery. JIA, NJO, OIU and IIM were also involved in patient care. GUE, OIU and IIM worked on sending the specimen for pathologic analysis, assisted in the drafting of the manuscript, performed PubMed research, and helped to critically revise the manuscript. SOU, JIA and NJO performed the gynaecological workup of the patient, assisted in the writing of the manuscript, and performed PubMed research. All authors read and approved the final manuscript.
